# Recommended Minimum Elements for Transparent Reporting of Multi-Jurisdiction Algorithm Feasibility Studies

**DOI:** 10.23889/ijpds.v9i5.2706

**Published:** 2024-09-18

**Authors:** Naomi Hamm, Sharon Bartholomew, Yinshan Zhao, Sandra Peterson, Saeed Al-Azazi, Kimberlyn McGrail, Lisa Lix

**Affiliations:** 1 University of Manitoba; 2 Public Health Agency of Canada; 3 Population Data BC; 4 University of British Columbia

## Background

Research and surveillance using routinely collected health data rely on algorithms to ascertain disease cases or health measures. Where algorithm validation studies are not possible due to lack of a reference standard, algorithm feasibility studies can be used to create and assess algorithms for use in more than one population or jurisdiction. Publication of the methods used to conduct feasibility studies is critical for transparency and reproducibility. Existing guidelines applicable to feasibility studies, including the STrengthening the Reporting of OBservational studies in Epidemiology (STROBE) and REporting of studies Conducted using Observational Routinely collected health Data (RECORD) statements, may benefit from additional elements to capture aspects particular to multi-jurisdiction algorithm feasibility studies and ensure full reproducibility.

## Methods

A subcommittee of members from Health Data Research Network (HDRN) Canada’s Algorithms and Harmonized Data Working Group (AHD-WG) reviewed items within the STROBE and RECORD guidelines and compared these to published feasibility studies. Items not contained within STROBE or RECORD but recommended to ensure transparent reporting of feasibility studies were identified. The AHD-WG reviewed and approved these additional recommended elements.

## Results

Eleven additional recommended elements were identified: one element for the title and abstract, one in the introduction, five in the methods, and four in the results sections. Elements primarily addressed reporting jurisdictional data variabilities, data harmonization methods, and algorithm implementation.

## Significance

Implementation of these recommended elements, alongside the RECORD guidelines, is intended to encourage consistent publication of methods that support reproducibility, as well as increase comparability and international collaborations.

